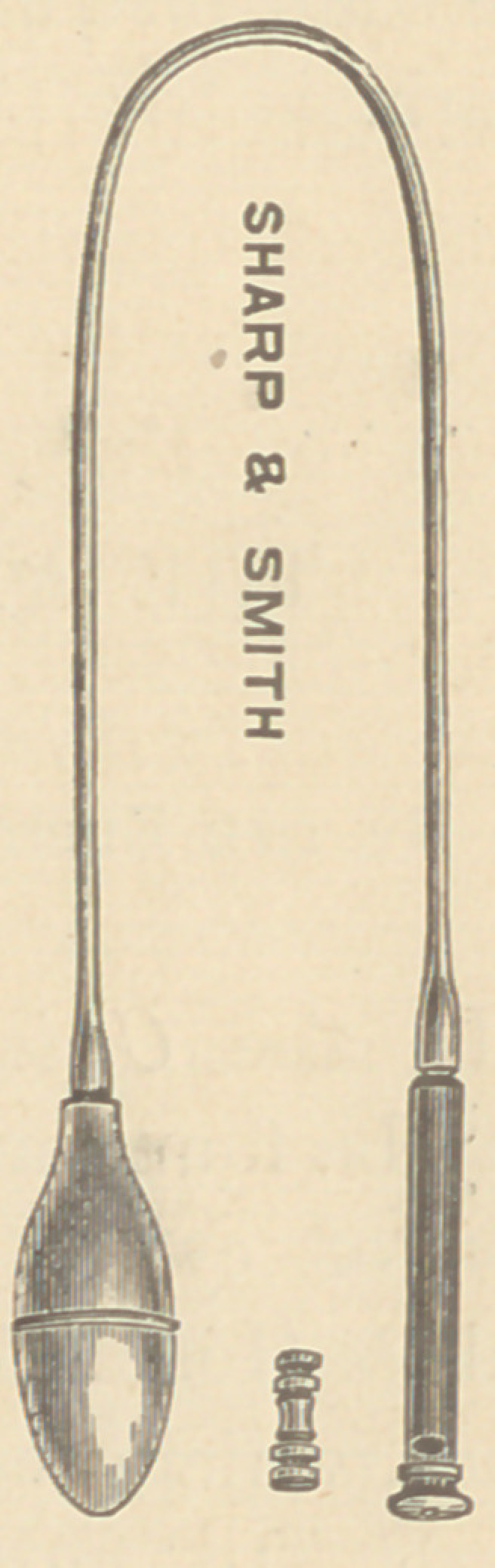# The Enballometer

**Published:** 1877-04

**Authors:** E. Fletcher Ingals

**Affiliations:** Lecturer on Diseases of the Chest and Physical Diagnosis, in the Spring Faculty of Rush Medical College, Chicago


### THE ENBALLOMETER.

By E. FLETCHER INGALS, M. D.

[Lecturer on Diseases of the Chest and Physical Diagnosis, in the Spring Faculty of Rush Medical College, Chicago.]

Auscultatory percussion was first brought to the notice of the profession by Drs. Camman and Clark, nearly forty years ago.

Their method of performing it was to press the objective end of a solid stethoscope* evenly on the surface directly over the most superficial portion of the organ or tumor to be examined, while the ear was applied to the opposite extremity of the instrument. At the same time an assistant performed percussion in the usual way, one or two inches from the point at which the stetherscope was applied.

* This instrument consists of a cylindrical piece of wood, about six inches long, and nearly an inch in diameter. The objective end is a truncated wedge, which allows of its easy application between the ribs. The other extremity is furnished with an ear-piece, which excludes external sounds

By this method, the examiner could map out the size of the liver, spleen, kidneys, heart or intra-thoracic tumors with great celerity, and almost as much precision as though the organs were exposed to view.

Notwithstanding the advantages offered by this method, it has never come into general use, probably on account of the inconvenience of obtaining assistants who would perform percussion satisfactorily. The Enballometer removes this difficulty by enabling the examiner to perform percussion for himself.

The instrument, as shown in the cut, consists of a hollow cylinder, three inches in length, with a diameter of seven-sixteenths of an inch. A chest piece is screwed upon the pectoral end of this cylinder, and by the other extremity it is connected, by means of a small rubber tube, eighteen inches in length, with a rubber bulb. Within the cylinder, fitting it loosely, plays a small, metallic plunger, or hammer. This hammer, shown separately in the cut, is turned from a rod of brass, is about one inch in length, and weighs half an ounce. Soft rubber cushions are placed within the cylinder at each end, to prevent the clacking sound which would otherwise be produced by the stroke of the hammer.

Immediately above the chest-piece, the cylinder is perforated, to allow free ingress and egress of air.

When the bulb is compressed, the hammer strikes upon the pectoral end of the instrument, and when it is allowed to expand, the hammer is thrown back by atmospheric pressure.

In using this instrument, the examiner applies the stethoscope with one hand over the most prominent part of the organ to be examined, while, with the other hand, he holds the enballometer, grasping the cylinder with the thumb and fore finger, and holding the rubber bulb in the palm of the hand by the remaining fingers.

If preferable, the examiner may hold the bulb with the disengaged fingers of the hand which holds the stethoscope.

The instrument should be held perpendicularly to the surface, and percussion made by sudden contraction and relaxation of the fingers holding the bulb. The relaxation should follow the contraction instantaneously, so that the hammer may at once recoil.

The results of the auscultatory percussion, aided by this instrument, are very satisfactory. The ordinary binaural stethoscope, with a small chest-piece, may be employed in place of the solid instrument recommended by Doctors Camman and Clark.

## Figures and Tables

**Figure f1:**